# The role of individual exopolysaccharides in antibiotic tolerance of *Pseudomonas aeruginosa* aggregates

**DOI:** 10.3389/fmicb.2023.1187708

**Published:** 2023-06-02

**Authors:** Ziwei Liang, Martin Nilsson, Kasper Nørskov Kragh, Ida Hedal, Júlia Alcàcer-Almansa, Rikke Overgaard Kiilerich, Jens Bo Andersen, Tim Tolker-Nielsen

**Affiliations:** ^1^Costerton Biofilm Center, Department of Immunology and Microbiology, University of Copenhagen, Copenhagen, Denmark; ^2^Bacterial Infections: Antimicrobial Therapies Group, Institute for Bioengineering of Catalonia (IBEC), The Barcelona Institute of Science and Technology (BIST), Barcelona, Spain

**Keywords:** biofilm, *Pseudomonas aeruginosa*, aggregates, extracellular matrix, antibiotic tolerance

## Abstract

The bacterium *Pseudomonas aeruginosa* is involved in chronic infections of cystic fibrosis lungs and chronic wounds. In these infections the bacteria are present as aggregates suspended in host secretions. During the course of the infections there is a selection for mutants that overproduce exopolysaccharides, suggesting that the exopolysaccharides play a role in the persistence and antibiotic tolerance of the aggregated bacteria. Here, we investigated the role of individual *P. aeruginosa* exopolysaccharides in aggregate-associated antibiotic tolerance. We employed an aggregate-based antibiotic tolerance assay on a set of *P. aeruginosa* strains that were genetically engineered to over-produce a single, none, or all of the three exopolysaccharides Pel, Psl, and alginate. The antibiotic tolerance assays were conducted with the clinically relevant antibiotics tobramycin, ciprofloxacin and meropenem. Our study suggests that alginate plays a role in the tolerance of *P. aeruginosa* aggregates toward tobramycin and meropenem, but not ciprofloxacin. However, contrary to previous studies we did not observe a role for Psl or Pel in the tolerance of *P. aeruginosa* aggregates toward tobramycin, ciprofloxacin, and meropenem.

## Introduction

Bacteria in biofilms display increased tolerance toward antibiotics and immune defenses, and as a consequence microbial biofilms are causing a multitude of persistent infections (Costerton et al., 1999; Hoiby et al., 2011). Since our current treatment regimens in many cases fail to cure biofilm-based infections, new anti-biofilm drugs and novel treatment strategies are urgently needed. Therefore, an understanding of the biological processes that are involved in the development of biofilm-associated antibiotic tolerance is essential.

Most of our knowledge about the molecular mechanisms underlying biofilm formation and biofilm-associated antibiotic tolerance originates from work with the bacterium *Pseudomonas aeruginosa* (Tolker-Nielsen, 2014). This bacterium is involved in a variety of biofilm-based infections, such as cystic fibrosis (CF) pneumonia, chronic wound infections, catheter-associated urinary tract infections, and ventilator-associated pneumonia, and accordingly *P. aeruginosa* has become a model organism for the study of biofilm formation and chronic infections (Tolker-Nielsen, 2014). Work done mainly with surface-associated biofilms has suggested that a number of different mechanisms play a role in the tolerance of biofilms to antibiotics. These mechanisms include (i) restricted penetration of antibiotics, (ii) the presence of bacterial subpopulations with low metabolic activity, and (iii) expression of specific genes that promote antibiotic tolerance (Hall and Mah, 2017; Ciofu and Tolker-Nielsen, 2019).

*Pseudomonas aeruginosa* can synthesize three different exopolysaccharides designated Pel, Psl, and alginate, although some strains produce only a subset of these exopolymers (Hoiby et al., 1987; Friedman and Kolter, 2004b; Jackson et al., 2004; Harmsen et al., 2010). During the course of CF lung infection there is a strong selection for *P. aeruginosa* mutants that overproduce biofilm matrix components. Overproduction of alginate by *P. aeruginosa mucA* mutants enables the bacteria to develop persistent infections in the lungs of CF patients (Hoiby, 1977; Lam et al., 1980). Overproduction of Psl and Pel by *P. aeruginosa* mutants such as *wspF* and *yfiR* also confer a benefit to the bacteria during CF lung infection (Starkey et al., 2009; Malone et al., 2010; Evans, 2015). *P. aeruginosa* mutants that overproduce Psl and Pel exopolysaccharide also show enhanced persistence in chronic wounds (Gloag et al., 2019).

When *P. aeruginosa* infects CF lungs or chronic wounds the bacteria are present in the form of aggregates embedded in the mucus or wound bed (Hoiby, 1977; Lam et al., 1980; Bjarnsholt et al., 2009; Fazli et al., 2009). It has been demonstrated that the physiology of the bacteria in aggregates is similar to the physiology of bacteria in surface-associated biofilms, and aggregates are therefore considered to be biofilms (Alhede et al., 2011). In the present study, we developed an antibiotic tolerance assay where *P. aeruginosa* grow as aggregates suspended in a thin layer of agar. Unlike surface-attached biofilms, the formation of agar-embedded aggregates does not require exopolysaccharide synthesis by the bacteria (Staudinger et al., 2014). The aggregate-based antibiotic tolerance assay therefore allows investigations of the role of individual exopolysaccharides in antibiotic tolerance, as aggregates of exopolysaccharide-producing bacteria can be compared to aggregates of bacteria that do not synthesize exopolysaccharide. We employed the aggregate antibiotic tolerance assay on a set of *P. aeruginosa* strains that were genetically engineered to over-produce a single, none, or all of the exopolysaccharides Pel, Psl, and alginate. This provided evidence that alginate, but not Psl or Pel, plays a role in the tolerance of *P. aeruginosa* aggregates toward tobramycin and meropenem, whereas none of the three exopolysaccharides play a role in the tolerance of the aggregates to ciprofloxacin.

## Materials and methods

### Bacterial strains and growth medium

The bacterial strains and plasmids used in this study are listed in Tables 1, 2, respectively. *P. aeruginosa* cultures were grown in LB medium (5 g yeast extract, 10 g tryptone, and 10 g NaCl per liter) at 37°C. LB supplemented with 1.5% agar was used for growth on solid media and LB supplemented with 0.8% agar was used for cultivation of agar-embedded aggregates. ABtrace medium (Pamp and Tolker-Nielsen, 2007) supplemented with 10 mM citrate and 1.5% agar was used for strain construction. When appropriate, gentamicin at 60 μg/ml was used for *P. aeruginosa* cultivation, whereas chloramphenicol at 6 μg/ml or gentamicin at 10 μg/ml was used for *Escherichia coli* cultivation. LB supplemented with 1.5% agar and Congo Red at 40 μg/ml and Coomassie brilliant blue at 20 μg/ml (Romling et al., 1998) was used as indicator plates to indicate synthesis of Psl and Pel.

**TABLE 1:** Strain list.

Strains	Description	References
***Escherichia coli***		
DH5-α	F^–^ *endA1 glnV44 thi-1 recA1 relA1 gyrA96 deoR nupG purB20 φ80dlacZ*Δ*M15* Δ*(lacZYA-argF)*U169, hsdR17(r_k_^–^ m_k_^+^), λ^–^	Lab collection
HB101	*rec*A *thi pro leu hsd*RM^+^, Sm^R^	Kessler et al., 1992
S17-1 λ*pir*	Str^R^, Tet^R^, F- RP4-2-Tc::Mu *aph*A::Tn7 *rec*A pro λpir lysogen	Lab collection
S17-1	*recA pro* (RP4-2Tet::Mu aphA::Tn*7*)	Simon et al., 1982
***Pseudomonas aeruginosa***		
PAO1	*P. aeruginosa* reference strain	Stover et al., 2000
Δ*wspF*	*wspF* mutant of PAO1	Rybtke et al., 2012
Δ*wspFΔpelA*	*wspF, pelA* double deletion mutant of PAO1	Rybtke et al., 2012
Δ*wspF*Δ*pslBCD*	*wspF, pslBCD* double deletion mutant of PAO1	Rybtke et al., 2015
Δ*pelA*Δ*pslBCD*	*pelA, pslBCD* double deletion mutant of PAO1	Rybtke et al., 2020
Δ*wspF*Δ*pelA*Δ*pslBCD*	*wspF, pelA, pslBCD* triple deletion mutant of PAO1	Rybtke et al., 2020
Epol+	*wspF*, *mucA22* double mutant of PAO1, resulting in a Pel, Psl, and alginate overproducing strain	This study
Epol−	*wspF, pelA, pslBCD, algD* quadruple mutant of PAO1, resulting in a Pel, Psl, and alginate deficient strain	This study
Alg+	*wspF, pelA, pslBCD, mucA22* quadruple mutant of PAO1, resulting in an alginate overproducing strain	This study
Pel+	*wspF, pslBCD, algD* triple mutant of PAO1, resulting in a Pel overproducing strain	This study
Psl+	*wspF, pelA, algD* triple mutant of PAO1, resulting in a Psl overproducing strain	This study
*mucA*Epol−	*mucA22, wspF, pelA, pslBCD, algD* mutant of PAO1, resulting in a MucA, WspF, Pel, Psl, and alginate deficient strain	This study

**TABLE 2:** Plasmid list.

Plasmid	Description	References
pΔalgD	pDONRPEX18Gm based *algD* knockout vector, GmR	Goltermann and Tolker-Nielsen, 2017
pENTRmucA22	Knock-in vector creating the *mucA22* mutant gene, GmR	Liang et al., 2022
pRK600	Mobilization plasmid, CmR	Kessler et al., 1992

### Strain constructions

The Δ*wspFmucA* and Δ*wspFmucA*Δ*pelA*Δ*pslBCD* strains were constructed by introduction of the *mucA22* allele in strain PAO1 Δ*wspF* (Rybtke et al., 2012) and Δ*wspF*Δ*pelA*Δ*pslBCD* (Rybtke et al., 2020), respectively, using the allelic exchange plasmid pENTRmucA22 (Liang et al., 2022). The PAO1 Δ*wspF*Δ*pslBCD*Δ*algD*, Δ*wspF*Δ*pelA*Δ*algD*, and Δ*wspF*Δ*pelA*Δ*pslBCD*Δ*algD* mutants were constructed by knocking out *algD* in PAO1 Δ*wspF*Δ*pslBCD* (Rybtke et al., 2015), Δ*wspF*Δ*pelA* (Rybtke et al., 2012), and Δ*wspF*Δ*pelA*Δ*pslBCD* (Rybtke et al., 2020), respectively, using the allelic exchange plasmid pΔalgD (Goltermann and Tolker-Nielsen, 2017). The plasmids were transferred into *P. aeruginosa* by two-parental or tri-parental mating using the donor strain *E. coli* S17-1 (Simon et al., 1982), or DH5α and the helper strain HB101/pRK600 (Kessler et al., 1992). The protocol used for allelic replacement was essentially as described by Hmelo et al. (2015). Merodiploid strains were selected on ABtrace-agar supplemented with 10 mM citrate and 60 μg/ml gentamicin. Next, double crossover mutants were isolated by streaking merodiploid colonies onto NS-LB-agar plates [LB medium without NaCl (Andersen et al., 2021)] supplemented with 10% sucrose for SacB-based counter selection. The plates were incubated at 30°C. Sucrose resistant, gentamicin-sensitive, double cross-over mutants were isolated, and the deletion of *algD* was verified with PCR and sequencing using primers seq-F-algD (CATCAAGTTGGTATCAAGTG) and seq-R-algD (GGAACACGTGCGACGG). Constructed mutants with the *mucA22* allele were isolated by selecting colonies displaying a slimy, mucoid appearance, and were verified by PCR and sequencing using primers mucA22_seqF (AGATATCGCCACCGTGATGC) and mucA22_seqR (AGGTCGTACCAGGAAGCCAG).

### Determination of minimal inhibitory concentrations

Minimal inhibitory concentration (MIC) values were determined by the use of cation adjusted Mueller-Hinton broth (3.0 g/L Beef Extract, 17.5 g/L Acid Hydrolysate of Casein, 1.5 g/L Starch, 20 mg/L Ca^2+^, 10 mg/L Mg^2+^, dH_2_O) in accordance with the guidelines of the European Committee on Antimicrobial Susceptibility Testing (EUCAST). Overnight-cultivated bacterial cultures were diluted to 10^6^ CFU/ml, and 100 μl cultures were treated with 100 μl twofold series diluted tobramycin, meropenem and ciprofloxacin (from 32 to 0.0625 μg/ml) in 96-well microtiter plates. The MICs were recorded as the lowest concentration that inhibited the growth of the bacteria after incubation at 37°C for 20 hours.

### Aggregate cultivation and antibiotic tolerance assessment

*Pseudomonas aeruginosa* aggregates were prepared by using a modified protocol based on the study of Goltermann and Tolker-Nielsen (2017). The main difference between the new and old model was that the bacteria were embedded in thin three-layer agar gels cast in Petri dishes, as opposed to centimeter thick agar gels harbored in syringes. The three-layer agar model was implemented to ensure the formation of aggregates under more uniform oxygen conditions. The procedure was as follows: 22 ml 0.8% LB agar (corresponding to a 3.5 mm thick layer) was cast in standard Petri dishes and allowed to solidify. An overnight culture of the *P. aeruginosa* strain of interest was diluted in LB to OD = 0.0001, and 50 μl was added to 19 ml 0.8% LB agar (approximately 45°C), which was subsequently cast onto the first agar layer in the Petri dish (resulting in a 3 mm thick layer). After solidification, 3 ml 0.8% LB agar (corresponding to a 0.5 mm thick layer) was cast on top of the other two agar layers. Petri dishes with bacteria embedded in the three-layer agar plates were incubated for 16 h at 37°C. Subsequently, gel plugs were acquired from the plates by punching down through the agar with a 10 mm biopsy knife. The gel plugs (approximate volume of 550 μl) were separately transferred to Falcon tubes containing 3.45 ml 0.9% NaCl with or without tobramycin, ciprofloxacin, or meropenem, resulting in an end concentration of 15 μg tobramycin/ml, 2 μg ciprofloxacin/ml, and 15 μg meropenem/ml, respectively. Tubes with and without antibiotics were incubated for 3 h with 110 RPM shaking at 37°C. The plugs were then transferred to new tubes and washed three times with 10 ml 0.9% NaCl with 10 min shaking (80 RPM). After the last wash the tubes were stored at 4°C in 10 ml 0.9% NaCl overnight. Next day, the gel plugs were transferred to 2 ml tubes containing 950 μl 0.9% NaCl and two ceramic beads (diameter 6.35 mm) (Fisher Scientific). The gels plugs were homogenized by the use of a MagNA Lyser (Roche) two times at 6,000 RPM for 10 s. Thereafter the bacterial aggregates were disrupted using degassing for 5 min and sonication for 5 min in an ultra sonication bath (Branson 2510). The solution was transferred, together with 1 ml solution of rinsed material of the tubes to Falcon tubes containing 12.5 ml 0.9% NaCl. The samples were then serially diluted and spotted on LB agar plates to enumerate the viable bacteria.

### Imaging and microscopy

Colony morphology images were obtained as follows. The respective *P. aeruginosa* strains were streaked from a colony or spotted (5 μl) from an overnight culture onto Congo Red/Coomassie Blue agar plates and grown for 24 h at 37°C. Images were then acquired using a Nikon D3300 digital camera.

The shape and size of the aggregates formed in the antibiotic tolerance assay were evaluated using a Zeiss LSM 710 confocal laser scanning microscope (CLSM) (Zeiss, Germany) running ZEN 2.1 (Zeiss, Germany). The cells within the aggregates were stained by applying 50 μl of a solution of 3 mM Syto9 (Life Technologies, USA) on top of the agar. The stain was allowed to penetrate the agar for 30 min before imaging. The aggregates were imaged using a 488 nm laser for excitation with emission filters ranging from 495 to 550 nm, respectively. Aggregate morphology images were obtained with a 10x #0.4 air objective from the surface with a depth-range of 200 μm acquired as 3D stacks with steps of 10 μm in the z-direction. Three-dimensional aggregate morphology images and quantitative data were processed in Imaris 9.7 (Bitplane, Switzerland).

### Statistical analysis

Data were analyzed by one-way or two-way analysis of variance (ANOVA) followed by Tukey’s multiple comparisons tests. *p* < 0.05 is considered significant.

## Results

### Construction of *P. aeruginosa* mutants that over-produce a single, none, or all of the three exopolysaccharides Pel, Psl, and alginate

We used the DNA sequenced *P. aeruginosa* PAO1 wild-type strain (Stover et al., 2000) and isogenic mutant derivatives for our studies of the role of exopolysaccharides in aggregate-associated antibiotic tolerance. We have found that *P. aeruginosa* PAO1 strains from different laboratories display different levels of antibiotic tolerance in our assay (data not shown), emphasizing the importance of using isogenic strains. For construction of the exopolysaccharide-overproducing mutants, we used the fact that introduction of a *mucA22* mutation (inactivating the MucA anti-sigma factor) results in highly increased transcription of the alginate operon (Boucher et al., 1997), and that deletion of the *wspF* gene (eliminating the WspF repressor of the WspR diguanylate cyclase) results in increased levels of the second messenger c-di-GMP that positively regulates production of Pel and Psl (Hickman et al., 2005). The *mucA* and *wspF* mutations are clinically relevant since *P. aeruginosa* strains with such mutations are frequently isolated from CF patients (Lam et al., 1980; Starkey et al., 2009). Since it has been reported that c-di-GMP, in addition to exopolysaccharide synthesis, also regulates the activity of an efflux pump that play a role in antibiotic tolerance (Liao et al., 2013), we included the Δ*wspF* mutation in all the mutant strains employed in this study, to ensure that the c-di-GMP content was at the same level in all the strains.

**FIGURE 1:**
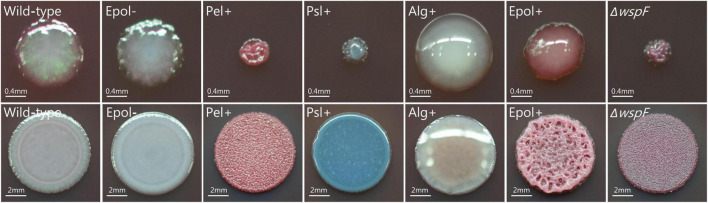
Colony morphology of the *P. aeruginosa* wild-type, Epol–, Pel+, Psl+, Alg+, Epol+, and Δ*wspF* strains. The respective *P. aeruginosa* strains were streaked **(upper row)** or spotted **(lower row)** on LB plates supplemented with Congo Red and Coomassie Blue, and images of representative colonies were acquired after 24 hours incubation at 37°C. Scale bars correspond to 0.4 mm **(upper row)** or 2 mm **(lower row)**.

To obtain strains that over-produce only Pel, Psl, or alginate we constructed the mutants Δ*wspF*Δ*pslBCD*Δ*algD*, Δ*wspF*Δ*pelA*Δ*algD*, and Δ*wspFmucA*Δ*pelA*Δ*pslBCD*, which were designated Pel+, Psl+, and Alg+, respectively. To obtain a strain unable to synthesize any of the polysaccharides we constructed a Δ*wspF*Δ*pelA*Δ*pslBCD*Δ*algD* mutant which was designated Epol−. To obtain a strain that overproduces Psl, Pel, and alginate, we constructed a *P. aeruginosa ΔwspFmucA* mutant which was designated Epol+. With this set of strains, we could compare the antibiotic tolerance of aggregates formed by *P. aeruginosa* bacteria that either over-produce a single of the three exopolysaccharides, do not produce any of the exopolysaccharides, or over-produce all three exopolysaccharides. The production of exopolysaccharides is known to affect the morphology of bacterial colonies, and accordingly the five constructed mutants all showed distinct colony morphologies on agar plates supplemented with Congo Red and Coomassie Blue. Figure 1 shows colonies initiated from single bacteria streaked from colonies (upper row), as well as colonies initiated from 5 μl spots of overnight cultures (lower row). The upper pictures provide information about colony size, while the lower pictures better illustrate the texture and color of the colonies. The Epol− strain formed white smooth colonies. The Pel+ strain formed small red and wrinkled colonies, whereas the Psl+ strain formed small blue and smooth colonies. The Alg+ and Epol+ colonies had a slimy mucoid appearance, but the Alg+ colonies were white whereas the Epol+ colonies were purple. The isogenic *P. aeruginosa* wild-type and Δ*wspF* mutant were included as controls, and the wild-type formed smooth and white colonies whereas the Δ*wspF* mutant formed small, wrinkled, purple colonies. The morphology of the colonies of the different strains on Congo Red/Coomassie Blue agar plates confirms that they all synthesize the expected polysaccharides (Hoiby, 1977; Romling et al., 1998; Friedman and Kolter, 2004a,b).

### Development of an assay for assessment of antibiotic tolerance of agar embedded *P. aeruginosa* aggregates

**FIGURE 2:**
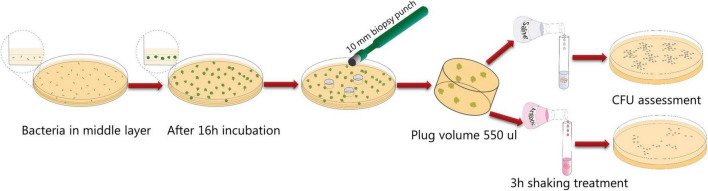
Schematic illustration of the aggregate antibiotic tolerance assay used in the current study.

We have previously used an antibiotic tolerance assay where *P. aeruginosa* bacteria grow as aggregates in centimeter thick LB agar gels harbored in syringes (Goltermann and Tolker-Nielsen, 2017). However, recently we have found that production of exopolysaccharides confer a high metabolic burden on the bacteria, and results in subsequent low metabolic activity if the bacteria are situated in a nutrient/oxygen limited environment (Lichtenberg et al., 2022). Since bacteria with low metabolic activity display increased antibiotic tolerance (Blair et al., 2015), we speculated that overproduction of exopolysaccharides under some conditions could affect antibiotic tolerance due to an affect on the metabolic state of the bacteria. Thus, a comparison between the antibiotic tolerance of a *P. aeruginosa* wild-type and an exopolysaccharide overproducing mutant might be affected by differential metabolic states in a system where a large part of the population is subject to oxygen limitation. Due to these considerations we developed a new assay for studying antibiotic tolerance where the *P. aeruginosa* aggregates are growing in a more homogenous environment than in our former assay. Initially we cultivated *P. aeruginosa* aggregates in a 3 mm thin LB agar layer cast in a petri dish. However, this agar layer has a high surface to volume ratio, and we found that a large proportion of the bacteria grew on the top and button of the agar and spread out on the agar-air and agar-plastic interfaces, ruining the experiments. Our solution to this was to use a system where the 3 mm thick LB agar layer with *P. aeruginosa* aggregates is sandwiched between a sterile agar layer at the bottom and a sterile agar layer at the top. We used a 0.5 mm thick top layer and a 3.5 mm thick bottom layer to facilitate sampling of agar plugs by the use of a punch biopsy knife (Figure 2).

**FIGURE 3:**
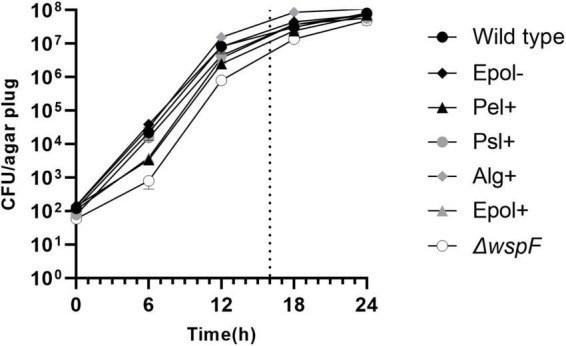
Curves showing the growth of the *P. aeruginosa* wild-type, Epol–, Pel+, Psl+, Alg+, Epol+, and Δ*wspF* aggregates in our assay. Equal numbers of bacteria from overnight cultures of the *P. aeruginosa* wild-type, Epol+, Epol–, Pel+, Psl+, Alg+, and Δ*wspF* strains were added to liquid agar and cast in petri dishes. The bacteria subsequently formed aggregates in the agar gels during incubation at 37°C, and at time intervals defined agar gel volumes were acquired from the agar plates by the use of a punch biopsy knife, and the number of colony forming units (CFU) per agar plug was determined by disintegration of the agar plugs and plating of the bacteria on agar plates followed by incubation. Average and standard deviation of three replicates are shown. The dotted line indicates the time point for antibiotic treatment in the antibiotic tolerance assay.

Initially we characterized the ability of each of our mutant strains to form aggregates in the LB agar gels. Equal numbers of bacteria from overnight cultures of the *P. aeruginosa* wild-type, Epol+, Epol−, Pel+, Psl+, Alg+, and Δ*wspF* strains were added to liquid agar and cast in Petri dishes (the wild-type and Δ*wspF* strains serving as controls). The bacteria subsequently formed aggregates in the agar gels during incubation at 37°C, and at time intervals defined agar gel volumes were acquired from the agar plates by the use of a punch biopsy knife (Figure 2). The number of colony forming units (CFUs) in each of the agar plugs was determined by disintegration of the agar plugs and plating of the bacteria on agar plates followed by incubation. As shown in Figure 3, the growth of the wild-type, Epol+, Epol−, Pel+, Psl+, Alg+, and Δ*wspF* strains were similar and they all entered stationary phase after around 24 h of growth.

**FIGURE 4:**
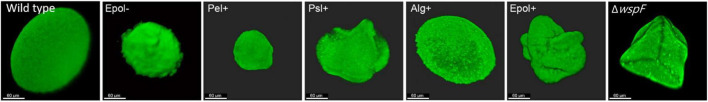
Morphology of aggregates formed by the *P. aeruginosa* wild-type, Epol–, Pel+, Psl+, Alg+, Epol+, and Δ*wspF* strains. The respective *P. aeruginosa* strains were grown as agar-embedded aggregates for 20 hours, upon which they were stained with Syto9 and images of representative aggregates were acquired by CLSM. Scale bars correspond to 60 μm.

We used CLSM to characterize the aggregates in the agar gels after 20 h of growth. Figure 4 shows representative images of the architecture and size of the aggregates. The aggregates had distinct morphologies, and the mutant aggregates (especially Pel+) appeared to be smaller than the wild-type aggregates. However, the curves shown in Figure 3 indicates that all aggregates contained around 5 × 10^5^ bacteria after 20 h of incubation. This estimate is based on the following assumptions: Each inoculum was carefully whirly mixed so the majority of the bacteria appeared as single cells at the time of inoculation (confirmed by microscopy). The aggregates were formed by growth of immobilized bacteria, since the agar (0.8%) restricted motility of the bacteria [Staudinger et al., 2014 demonstrated that the motility of *P. aeruginosa* is restricted in 0.8% agar]. Accordingly, each bacterium present in the agar at time zero grew and formed one aggregate. Since the CFU per agar plug was approximately 100 at time zero, the number of aggregates per agar plug was around 100 (at all time points). This means that after 20 h of growth the CFU per aggregate was around 5 × 10^5^ (corresponding to 5 × 10^7^ CFU/agar plug divided by 100 aggregates/agar plug).

### Aggregate-associated antibiotic tolerance of the *P. aeruginosa* mutants and wild-type

**FIGURE 5:**
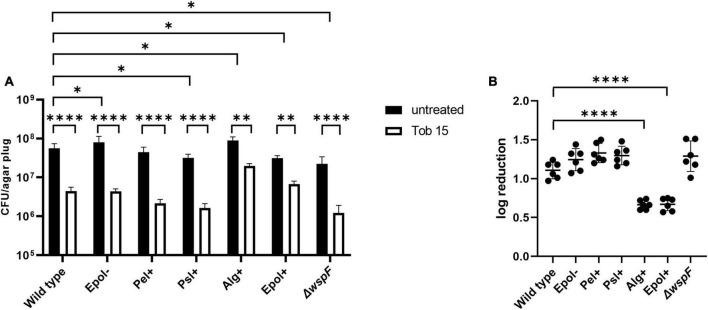
Tolerance to tobramycin of aggregates formed by the *P. aeruginosa* wild-type, Epol–, Pel+, Psl+, Alg+, Epol+, and Δ*wspF* strains. The respective *P. aeruginosa* strains were grown as agar-embedded aggregates for 16 h. Subsequently, agar plugs containing agar-embedded aggregates were obtained from the agar plates using a punch biopsy knife. The agar plugs were then treated for 3 h with either 15 μg/ml tobramycin or saline. Next, the antibiotic was removed by a washing procedure and the agar plugs were disintegrated, serially diluted and spotted on LB agar plates and incubated for enumeration of the surviving bacteria **(A)** and calculation of fold reduction mediated by the antibiotic treatment **(B)**. Panel **(A)** shows averages and standard deviations of six replicates, and the significance (two-way ANOVA) of the difference between the CFU values of select groups are indicated by stars: *p < 0.05; ^**^p < 0.01; ^****^p < 0.0001. The significance (one-way ANOVA) of the difference between the log reduction values of the wild-type and the other strains are indicated by stars in panel **(B)**: ^****^p < 0.0001.

**FIGURE 6:**
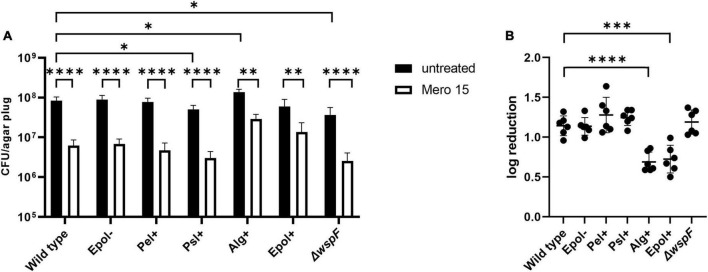
Tolerance to meropenem of aggregates formed by the *P. aeruginosa* wild-type, Epol–, Pel+, Psl+, Alg+, Epol+, and Δ*wspF* strains. The respective *P. aeruginosa* strains were grown as agar-embedded aggregates for 16 h. Subsequently, agar plugs containing agar-embedded aggregates were obtained from the agar plates using a punch biopsy knife. The agar plugs were then treated for 3 h with either 15 μg/ml meropenem or saline. Next, the antibiotic was removed by a washing procedure and the agar plugs were disintegrated, serially diluted and spotted on LB agar plates and incubated for enumeration of the surviving bacteria **(A)** and calculation of fold reduction mediated by the antibiotic treatment **(B)**. Panel **(A)** shows averages and standard deviations of six replicates, and the significance (two-way ANOVA) of the difference between the CFU values of select groups are indicated by stars: *p < 0.05; ^**^p < 0.01; ^****^p < 0.0001. The significance (one-way ANOVA) of the difference between the log reduction values of the wild-type and the other strains are indicated by stars in panel **(B)**: ^***^p < 0.001; ^****^p < 0.0001.

**FIGURE 7:**
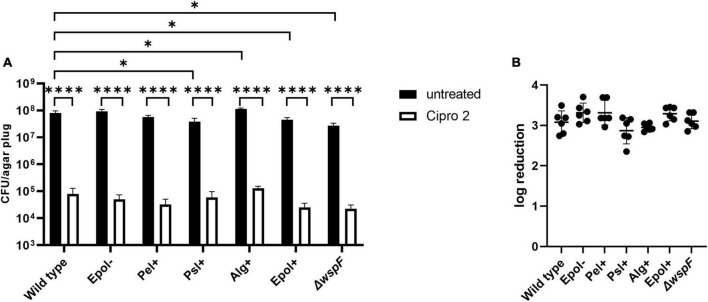
Tolerance to ciprofloxacin of aggregates formed by the *P. aeruginosa* wild-type, Epol–, Pel+, Psl+, Alg+, Epol+, and Δ*wspF* strains. The respective *P. aeruginosa* strains were grown as agar-embedded aggregates for 16 h. Subsequently, agar plugs containing agar-embedded aggregates were obtained from the agar plates using a punch biopsy knife. The agar plugs were then treated for 3 h with either 2 μg/ml ciprofloxacin or saline. Next, the antibiotic was removed by a washing procedure and the agar plugs were disintegrated, serially diluted and spotted on LB agar plates and incubated for enumeration of the surviving bacteria **(A)** and calculation of fold reduction mediated by the antibiotic treatment **(B)**. Panel **(A)** shows averages and standard deviations of six replicates, and the significance (two-way ANOVA) of the difference between the CFU values of select groups are indicated by stars: *p < 0.05; ^****^p < 0.0001. There was no significant difference between the log reduction values shown in panel **(B)**.

We chose the antibiotics tobramycin, ciprofloxacin and meropenem for our investigation of the role of exopolysaccharides in antibiotic tolerance of *P. aeruginosa* aggregates, since these antibiotics have distinct chemical features and mode of action, and are often used for the treatment of *P. aeruginosa* biofilm infections (Ciofu et al., 2015). We used the concentrations 15 μg/ml for tobramycin, 15 μg/ml for meropenem, and 2 μg/ml for ciprofloxacin, which correspond to 15 × MIC for tobramycin, 15 × MIC for meropenem, and 8 × MIC for ciprofloxacin (Table 3 shows MIC values determined for all strains according to EUCAST guidelines). The *P. aeruginosa* strains were grown as agar-embedded aggregates for 16 h, and gel plugs with the aggregates were then treated for 3 h with antibiotics, or with 0.9% NaCl as the non-treatment control (Figure 2). Subsequently, the antibiotics were removed by a washing procedure and the gel plugs and aggregates were disintegrated and the CFUs and fold reduction mediated by the antibiotics were determined. The aggregates formed by the Alg+ and Epol+ strains showed about half a log higher tolerance to tobramycin and meropenem than the aggregates formed by the wild-type, Epol−, Pel+, and Psl+ strains (Figures 5, 6). However, all the tested strains showed the same level of aggregate-associated tolerance toward ciprofloxacin (Figure 7). This suggests that alginate, but not Psl or Pel, plays a role in the tolerance of the aggregates toward tobramycin and meropenem, whereas none of the three exopolysaccharides play a role in the tolerance of the aggregates toward ciprofloxacin.

**TABLE 3:** Minimal inhibitory concentration values.

Strain	MIC (μg/ml)		
	Tobramycin	Meropenem	Ciprofloxacin
Wild-type	1	1	0.25
Δ*wspF*	1	1	0.25
Epol−	1	1	0.25
Pel+	1	1	0.25
Psl+	1	1	0.25
Alg+	1	1	0.25
Epol+	1	1	0.25

The CFUs of the untreated samples for some of the strains deviate slightly but significantly from the CFU of the untreated samples of the wild-type (Figures 5–7). However, we do not think this is critical for the experiments, since each of the treated samples have their own non-treatment control. Importantly, there is no correlation between the CFUs of the untreated samples and the antibiotic tolerance of the aggregates. For example, the CFU of the untreated samples of the Alg+ strain is higher than that of the wild-type, whereas the CFU of the untreated samples of the Epol+ strain is lower than that of the wild-type; but the Alg+ and Epol+ aggregates are both more tolerant to tobramycin/meropenem than the wild-type aggregates.

**FIGURE 8:**
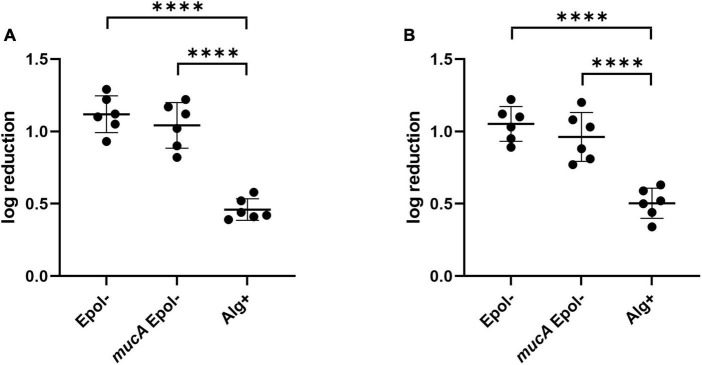
Tolerance to tobramycin **(A)** and meropenem **(B)** of aggregates formed by the *P. aeruginosa* Epol–, *mucA*Epol–, and Alg+ strains. The respective *P. aeruginosa* strains were grown as agar-embedded aggregates for 16 h. Subsequently, agar plugs containing agar-embedded aggregates were obtained from the agar plates using a punch biopsy knife. The agar plugs were then treated for 3 h with either 15 μg/ml tobramycin **(A)** or 15 μg/ml meropenem **(B)**, or saline. Next, the antibiotic was removed by a washing procedure and the agar plugs were disintegrated, serially diluted and spotted on LB agar plates and incubated for enumeration of the surviving bacteria and calculation of fold reduction mediated by the antibiotic treatment. The significance (one-way ANOVA) of the difference between the log reduction values of the Epol– strain and the other strains are indicated by stars: ^****^p < 0.0001.

To exclude that the increased tobramycin/meropenem tolerance of the Alg+ and Epol+ aggregates was due to a lack of the MucA protein *per se*, we constructed a Δ*wspFmucA*Δ*pelA*Δ*pslBCD*Δ*algD* mutant (*mucA*Epol−), and assessed the tolerance of *mucA*Epol− aggregates toward tobramycin and meropenem. As shown in Figure 8, the tolerance of the *mucA*Epol− aggregates was at the same level as the tolerance of the Epol− aggregates, but lower than the tolerance of the Alg+ aggregates. This indicates that the increased tolerance of the Alg+ and Epol+ aggregates is caused by the presence of the alginate, and not by a pleotropic effect of the *mucA* deletion.

## Discussion

The presence of bacterial aggregates suspended in host secretions is a characteristic feature of *P. aeruginosa* infections in CF lungs (Hoiby, 1977; Lam et al., 1980) and chronic wounds (Fazli et al., 2009). Accordingly, experimental setups supporting growth of aggregates are relevant for investigations aimed at elucidating the mechanistic basis of biofilm-associated antibiotic tolerance. Since exopolysaccharides are not needed for the formation of agar-embedded aggregates, the aggregate-based assay enables assessment of the role of individual exopolysaccharides in antibiotic tolerance in comparison to mutant strains that do not produce any exopolysaccharides. Accordingly, the original aim of the current study was to investigate the role of individual *P. aeruginosa* exopolysaccharides in aggregate-associated antibiotic tolerance. To this end, we constructed a set of *P. aeruginosa* PAO1 mutants that were genetically engineered to over-produce a single, none, or all of the exopolysaccharides Pel, Psl, and alginate. Recently, however, we have found that production of exopolysaccharides confer a high metabolic burden on bacteria, resulting in subsequent low metabolic activity if the bacteria are situated in a nutrient/oxygen limited environment (Lichtenberg et al., 2022). Therefore, in the current study we developed an aggregate tolerance assay where the aggregates are expected to grow under uniform oxygen and nutrient conditions. In support of this, we found that the aggregates of all the strains grew equally well in our assay, and that all strains entered stationary phase at around the same time point.

We chose to perform antibiotic treatment of aggregates that had been growing for 16 h in our model. At this time point the aggregates contained around 10^5^ bacteria, and the bacteria in the aggregates were growing, although their growth rate was beginning to decline. We think these conditions likely are clinically relevant, as evidence for limited growth of *P. aeruginosa* in CF lungs has been reported (Yang et al., 2008; Kragh et al., 2014). The antibiotic tolerance assays were conducted with the antibiotics tobramycin, ciprofloxacin, and meropenem which are relevant for the treatment of *P. aeruginosa* lung infections (Ciofu and Tolker-Nielsen, 2019). In agreement with previous investigations (Staudinger et al., 2014; Goltermann and Tolker-Nielsen, 2017) we found that aggregates formed by a mutant deficient in exopolysaccharide synthesis (Epol−) displayed the same antibiotic tolerance as wild-type aggregates. Moreover, we found that aggregates formed by mutants that overproduce alginate (Alg+ and Epol+) showed about half a log increased tolerance to tobramycin and meropenem, but not increased tolerance to ciprofloxacin. Furthermore, we found that mutants that overproduce Pel (Pel+) or Psl (Psl+) did not display increased tolerance to tobramycin, meropenem and ciprofloxacin in comparison to wild-type aggregates. This suggested in contrast to a number of other studies (discussed below) that Psl and Pel do not play a direct role in antibiotic tolerance of *P. aeruginosa* aggregates.

Using an aggregate antibiotic tolerance assay where growth is expected to occur under more heterogeneous oxygen conditions than in our new assay, we have previously found that aggregates of a *P. aeruginosa mucA* mutant (overproducing alginate, producing Psl and Pel) were 50-fold more tolerant to tobramycin than wild-type aggregates, and sixfold more tolerant to ciprofloxacin than wild-type aggregates (Goltermann and Tolker-Nielsen, 2017). Moreover we found in our previous study that aggregates of a *P. aeruginosa ΔwspF* mutant (overproducing Psl and Pel) were 30-fold more tolerant to tobramycin than wild-type aggregates, and fourfold more tolerant to ciprofloxacin than wild-type aggregates (Goltermann and Tolker-Nielsen, 2017). Since evidence has been provided that the *P. aeruginosa* biofilm matrix blocks the penetration of tobramycin into the biofilm, but is not a barrier for ciprofloxacin (Stewart, 1996; Walters et al., 2003; Tseng et al., 2013), we suggested in our former study that the presence of the matrix components may alter the physiology of the bacteria in the aggregates (Goltermann and Tolker-Nielsen, 2017).

In an early stage of our present work we hypothesized that the combination of Psl and Pel (as produced by the Δ*wspF* mutant) might give other antibiotic tolerance properties than the individual polysaccharides (as produced by the Psl+ and Pel+ mutants). However, when we included the Δ*wspF* mutant in the present study we found that the Δ*wspF* aggregates were not more tolerant to antibiotics than the Psl+, Pel+, and wild-type aggregates.

We believe that the contrasting results between our present and former (Goltermann and Tolker-Nielsen, 2017) findings might be due to differences in oxygen conditions in the two aggregate-based antibiotic tolerance assays. In support of this view, we have recently reported that production of exopolysaccharides confer a high metabolic burden on bacteria, and results in subsequent low metabolic activity if the bacteria are situated in a nutrient/oxygen limited environment (Lichtenberg et al., 2022). Since bacteria with low metabolic activity display increased antibiotic tolerance (Blair et al., 2015), a comparison between the antibiotic tolerance of a *P. aeruginosa* wild-type and an exopolysaccharide overproducing mutant might be affected by differential metabolic states in a model system where a large part of the population is subject to oxygen limitation.

If Pel and Psl synthesis can affect biofilm-associated antibiotic tolerance due to an effect on the metabolic state of the bacteria this might explain why varying results have been seen in previous studies using different assays. Colvin et al. (2011) used an assay with colony biofilms growing on a filter on agar plates, and found that Pel-overproducing *P. aeruginosa* strains displayed increased tolerance to tobramycin, but not to ciprofloxacin (Colvin et al., 2011). However, overproduction of Psl did not confer protection of the biofilms against tobramycin or ciprofloxacin in that study (Colvin et al., 2011). Billings et al. (2013) used MBEC microtiter plate assays and flow-chamber experiments, and demonstrated that young biofilms of a *P. aeruginosa* mutant strain lacking Psl production were more sensitive to tobramycin, ciprofloxacin, colistin and polymyxin than wild-type biofilms (Billings et al., 2013). Reduced antibiotic tolerance of the Δ*psl* mutant in comparison to the wild-type was only seen in young biofilms, but was not evident in biofilms grown for 48 and 72 h (Billings et al., 2013). Moreover, a Δ*pel* mutant showed similar antibiotic tolerance as the wild-type in 1-day-old biofilms, but a decreased tolerance, although modest, in 2-day-old biofilms (Billings et al., 2013). Murakami et al. (2017) employed MBEC microtiter plate assays to assess the tolerance of a *P. aeruginosa* wild-type, Δ*psl* and Δ*pel* mutant, and found that both Psl and Pel plays a role in the tolerance of the biofilm to biapenem (Murakami et al., 2017).

Our studies indicate that alginate can protect biofilms against tobramycin and meropenem. In agreement with this, Hentzer et al. (2001), using flow cell experiments and a biofilm rotator system, demonstrated that alginate overproduction confer increased tobramycin tolerance of *P. aeruginosa mucA* biofilms compared to wild-type biofilms. In addition, Alipour et al. (2009) demonstrated that administration of alginate lyase and DNase enhanced the killing activity of tobramycin in *P. aeruginosa* biofilms.

The finding that the negatively charged alginate (Evans and Linker, 1973) protected *P. aeruginosa* aggregates against the positively charged tobramycin and the zwitterionic meropenem, but not the neutral ciprofloxacin, suggests a protection mechanism involving electrostatic repulsion. This suggestion is in accordance with the finding that the neutral Psl (Ma et al., 2007) and the positively charged Pel (Jennings et al., 2015) did not protect the aggregates against tobramycin, meropenem, or ciprofloxacin. It also aligns with earlier studies suggesting that the *P. aeruginosa* biofilm matrix is a barrier for tobramycin but not ciprofloxacin (Stewart, 1996; Walters et al., 2003; Tseng et al., 2013).

In conclusion, our study provides evidence that alginate can protect *P. aeruginosa* aggregates against antibiotics, which is in accordance with the strong selection for mucoid *P. aeruginosa* strains in CF patients that are routinely treated with antibiotics (Hoiby, 1977; Lam et al., 1980; Ciofu et al., 2015). In addition, our study suggests that Psl and Pel may not have a direct role in antibiotic tolerance. The selection for *P. aeruginosa* mutants that overproduce Pel and Psl in CF lung infections and chronic wounds (Starkey et al., 2009; Malone et al., 2010; Evans, 2015; Gloag et al., 2019), could instead be due to a role of Psl and Pel in immune evasion (Malone et al., 2010; Mishra et al., 2012).

## Data availability statement

The original contributions presented in this study are included in the article/supplementary material, further inquiries can be directed to the corresponding author.

## Author contributions

ZL, MN, KK, JA, and TT-N designed the experiments. ZL, MN, KK, IH, JA-A, and RK performed the experiments. ZL, JA, and TT-N interpreted the data. ZL and TT-N wrote the manuscript with input from all other authors. All authors contributed to the article and approved the submitted version.
